# Detecting 17 fine-grained dental anomalies from panoramic dental radiography using artificial intelligence

**DOI:** 10.1038/s41598-022-09083-2

**Published:** 2022-03-25

**Authors:** Sangyeon Lee, Donghyun Kim, Ho-Gul Jeong

**Affiliations:** 1 Department of Bio and Brain Engineering, Korea Advanced Institute of Science and Technology, Daejeon, 34141 Republic of Korea; 2 InVisionLab, Seoul, 05854 Republic of Korea; 3 Department of Advanced General Dentistry, Yeonsei University, Seoul, 03722 Republic of Korea

**Keywords:** Panoramic radiography, Medical imaging

## Abstract

Panoramic dental radiography is one of the most common examinations performed in dental clinics. Compared with other dental images, it covers a wide area from individual teeth to the maxilla and mandibular area. Dental clinicians can get much information about patients’ health. However, it is time-consuming and laborious to detect all signs of anomalies because these regions are very complicated. So it is needed to filter out healthy images to save clinicians’ time to examine. For this, we applied modern artificial intelligence-based computer vision techniques. In this study, we built a model to detect 17 fine-grained dental anomalies which are critical to patients’ dental health and quality of life. We used about 23,000 anonymized panoramic dental images taken from local dental clinics from July 2020 to July 2021. Our model can detect these abnormal signs and filter out normal images with high sensitivity of about 0.99. The result indicates that our model can be used in real clinical practice to alleviate the burden of clinicians.

## Introduction

Artificial intelligence techniques are fast developing and applied to various industries. Healthcare service is one of the most potential fields to take advantage of those techniques because of the fast accumulation of massive and complex features of medical data. Especially with the development of deep learning techniques, the convolutional neural network (CNN) model and its variances are used in many fields of image analysis, such as classification and object detection. Because many medical data are collected in image formats, such as skin image datasets^1^ and ophthalmoscope^2^ images, artificial intelligence techniques are widely applied to various medical fields and tasks. Especially, regional convolutional neural network (RCNN) models are widely applied to the medical field in order to detect some medical signs from a given image to detect the region of breast cancer^3^, pneumonia^4^, and so on.

Dentistry is also one of the hospital branches where artificial intelligence techniques are vividly applied because it takes and uses a lot of medical images during a clinical routine. Patients admitted to hospitals are usually examined by radiographs. From simple intra-oral periapical x-rays to computed tomography, there are various tools to examine patients’ oral health and status. Panoramic dental radiography is one of the most commonly performed imaging techniques by dentists and oral surgeons in everyday practice. It shows good patient acceptance because it is simple and painless and also uses a small amount of radial dose^5^. Panoramic dental radiography produces an image that includes not only maxillary and mandibular dental arches, which are mainly examined by dentists, but also the surrounding structures as the maxillary sinus, nasal fossa, temporomandibular joints, styloid processes, and hyoid bone^6^. Radiographic findings from these structures are suggested for diagnostic features of many diseases from dental anomalies to systemic diseases like hypoparathyroidism, hyperparathyroidism, and osteoporosis^7^. However, orthodontic and surrounding areas shown in panoramic dental radiography are complicated regions so correct diagnoses of anomalies can be very laborious and time-consuming, also potentially inaccurate^8^. Also, dentists might only concentrate on teeth of symptoms and regions of interest due to lack of time.

One of the methods to reduce this burden is computer-aided anomaly detection techniques. Diagnosis of dental anomalies with computational analysis of panoramic dental radiography is not a novel concept. Before the recent development of deep learning techniques, scientists tried to diagnose dental anomalies through image texture calculations^9,10^ or abnormality thresholding^11^. And with the recent advances of deep learning techniques, it has been quickly applied to increase the performance of analyzing panoramic dental radiography. Many of the studies are focused on detecting some signs of dental diseases from panoramic dental radiography. Especially, a large proportion of previous studies mainly selected carious lesions as their detection targets^9–14^, and few studies are focused on another dental anomaly, such as periodontal bone loss^15^, odontogenic cyst, tumor^16^, osteoporosis^17^, impacted tooth^8^, and so on. These studies used variations of convolutional neural network models developed for image classification or object detection tasks, such as MobileNet V2^12^, single-column deep convolutional neural network (SC-DCNN)^17^, regional convolutional neural network (RCNN)^13^.

These previous studies could detect targeted disease with satisfying performance, however, most of the works targeted only one or a small number of diseases or abnormal signs. This limitation makes it hard to take one of the major advantages of panoramic dental radiography, which covers a wide area from individual teeth to mandibular and maxillary regions. Also, to reduce the burden of dental clinicians, it is needed to filter out healthy images to reduce the number of images that need to be manually examined.

Here, we selected fine-grained 17 anomalies that are closely related to patients’ quality of life and also can be detected from panoramic dental radiography. We categorized them into four groups: carious lesions, calcifications, anomalies in dental regions, and anomalies in surrounding regions. To distinguish the dental region and surrounding regions, we used the method suggested by Langland, et al.^18^. Features and objects shown in zone 1 are included in the formal category, while others shown in the rest part of the image, from zone 2 to zone 6, are included in the latter category.

A detailed list of anomalies, their main features, and criteria for labeling in the image are shown in Table 1. We built and trained a deep learning model to detect signs of those 17 diseases from an image. It covers a wide region of panoramic radiography and will reduce the burden of clinicians in hospitals and help the prevention and early diagnosis of diseases.

**Table 1:** Category, name, and labeling criteria of 17 fine-grained dental anomalies.

Category	Name of anomaly	Descriptions and diagnostics
Soft tissue calcification	Calcified carotid atherosclerotic plaque	Irregular linear radiopacity between mandibular angle or hyoid bone and cervical vertebrae
	Lymph node calcification	One or multiple irregular or cauliflower-like radiopacity in the lower or rear of the mandibular angle or between the mandibular and cervical vertebrae
	Ossification of the stylohyoid ligament	Long and thin radiopacity in anteroinferior direction between styloid process and hyoid bone
	Tonsillar calcification	One or multiple radiopacities in the dorsal surface of the tongue overlaps with the mandibular ramus
Carious lesions	Cervical caries or abrasion	Notch or half-moon shaped radiolucency in the cervical area of the tooth
	Dental caries or coronal defect	Various patterns of radiolucency in occlusal surface of the tooth
	Proximal caries	Various patterns of radiolucency in the interproximal surface of the tooth
	Secondary caries	Various patterns of radiolucency in the inferior area of restorations
Anomalies in the dental region	External root resorption	Irregular shape of the root
	Impacted tooth	A condition in which the tooth is not normally erupted and is ambushed in the bone, found in jaw
	Periapical radiolucency	Various patterns of radiolucency in the periapical area of the tooth
	Residual root	Loss of coronal portion in tooth
	Supernumerary tooth	Tooth in addition to the normal series of deciduous or permanent dentition
	Tooth overlapped with mandibular canal	The root of the third molar is overlapped with the mandibular canal
Anomalies in surrounding region	Mucosal thickening in maxillary sinus	Various patterns of radiopaque shadow in maxillary sinus
	Radiopacity in jaw	Various patterns of radiopacity in jaw
	Retention pseudocyst in maxillary sinus	Dome-shaped radiopaque shadow in maxillary sinus

## Result

### Dataset generation

**Figure 1:**
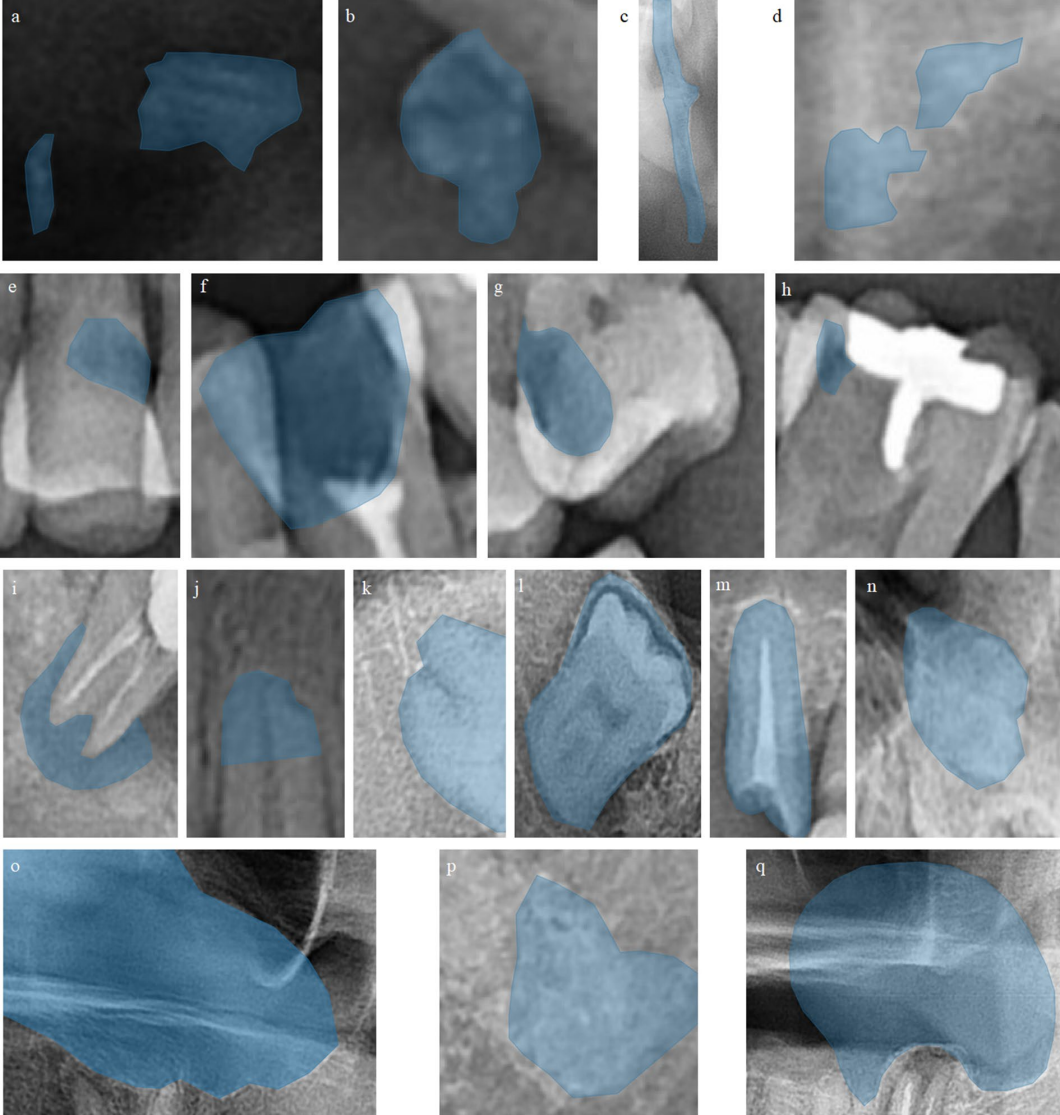
Examples of labeled 17 dental anomalies. **(a)** Calcified carotid atherosclerotic plaque, **(b)** lymph node calcification, **(c)** ossification of stylohyoid ligament, **(d)** tonsillar calcification, **(e)** cervical caries or abrasion, **(f)** dental caries or coronal defect, **(g)** proximal caries, **(h)** secondary caries, **(i)** periapical radiolucency, **(j)** external root resorption, **(k)** tooth overlapped with mandibular canal, **(l)** impacted tooth, **(m)** residual root, **(n)** supernumerary tooth, **(o)** mucosal thickening on maxillary sinus, **(p)** radiopacity in jaw, and **(q)** retention pseudocyst on maxillary sinus.

A large amount of high-quality datasets is one of the essential factors to take advantage of machine learning techniques. To build a model that is directly applicable to real clinical practices, we used panoramic dental images taken from local dental clinics. In this study, a total of 22,999 panoramic dental images were collected from 30 local dental clinics during a year, from July 2020 to July 2021. The size of image datasets used in previous studies varies from 87 to 3,000 according to the review^14^. Compared with those datasets, the size of our dataset exceeds the size of the others. The dataset is manually labeled by a dental radiography expert. Figure 1 shows some examples of labeled images.

### Anomaly detection

**Figure 2:**
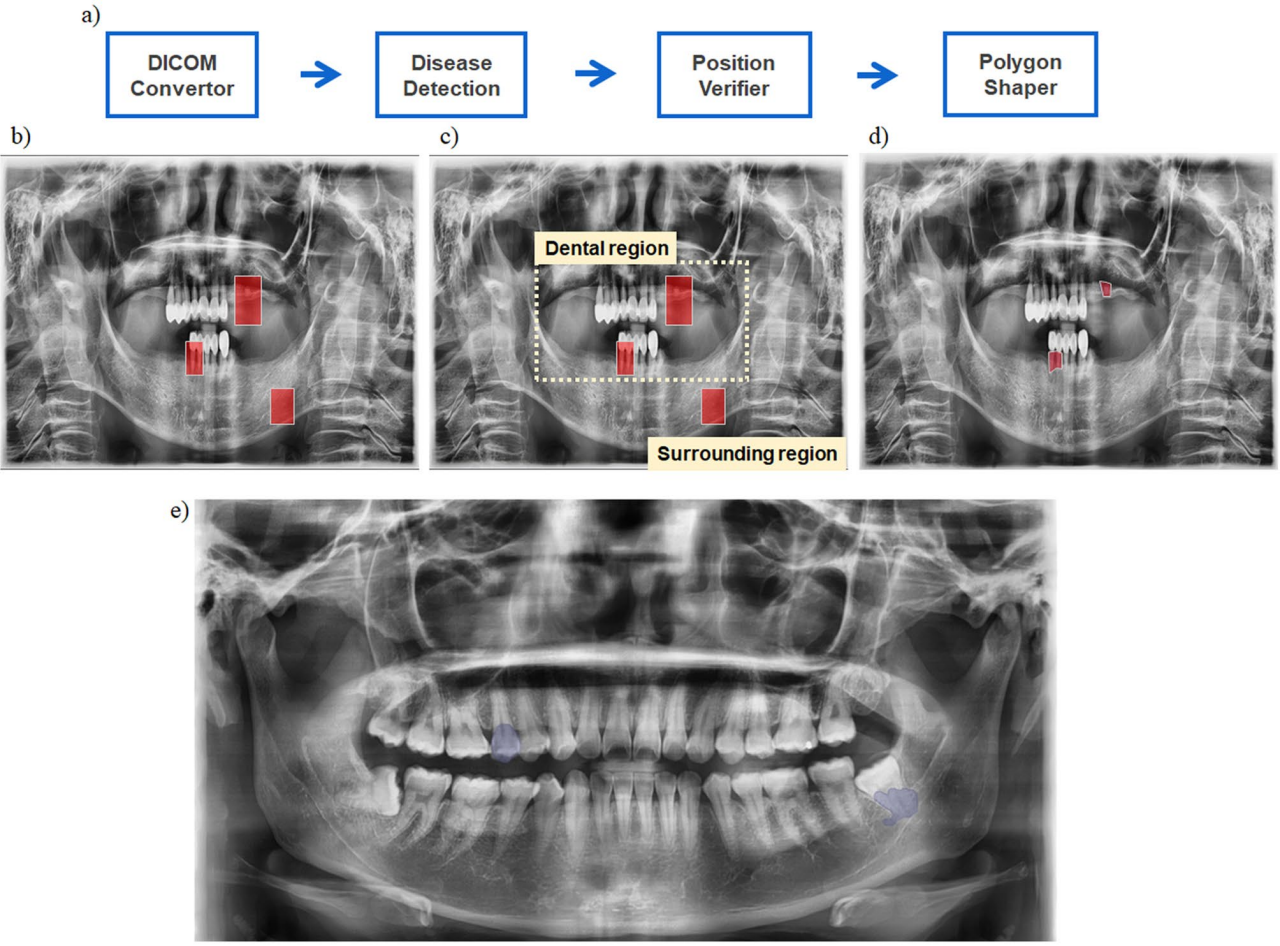
Anomaly detection process of our model. **(a)** Overall process of this model, **(b)** result of disease detection stage. Boxes are found from the faster R-CNN architecture. **(c)** Example of position verifier, carious lesion, for example, is known to be located in dental region, so boxes which are located in surrounding region are deleted. **(d)** Result of polygon shaper. To notice dental clinicians why the regions are detected to be abnormal, this model narrows down the region into polygon shape. **(e)** Example figure of final outcome of the model. Proximal caries in upper tooth is in asymptomatic stage but this model can find this. This model can contribute the early detection and diagnosis of several dental anomalies before they go severe, improving outcomes dental healthcare and patients’ quality of life.

Our detection model consists of four parts (Fig. 2a). First, we convert DICOM formatted images directly transferred from dental clinics into PNG format. Then it detects anomalies through trained faster R-CNN models^19^. Basically it is an object detection model, we got object boxed regions of high possibilities of anomalies (Fig. 2b). Next, we filter out some boxes that are not located in a predetermined region (Fig. 2c). For example, if detected boxes are about carious lesions, it is obvious that the boxes should be located in the dental region. However, if some of the boxes are not located in the region but in the surrounding region, we can be sure that those boxes are absurd, so we can filter out those boxes. In the final stage, we narrow down the region of abnormal signs from a box form to a polygon (Fig. 2d). Through this stage dental clinicians can get high-resolution information not only the location and the type of anomalies found in the image, also the specific regions that show the feature of the anomalies. We used a prebuilt library, Detectron2^20^, in the polygon shaper stage. Figure 2e is an example of our detection model. There are two signs of dental anomalies, proximal caries and periapical radiolucency. The proximal caries in the figure is in asymptotic stage so it may be stay undetected without careful examination. This model successfully detected it which means that the model can help early detection of selected anomalies.

We tested our detection model using part of our dataset. We divided images to training, validation and test datasets. Train and validation datasets are used to training phase and test dataset are used to evaluate our model. Table 2 shows the class-wise performance of our detection model applied to the test dataset and Table 3 contains information about detailed numbers of test datasets. Our model shows very high specificity, in most classes over 95%, which means it can filter out most of the normal or healthy images. High specificity means that this model can be used to reduce the burden of examination of dental clinicians because it successfully filters out healthy images so that clinicians can focus on other images. Precision and sensitivity vary depending on the type of anomalies but generally the score exceeds or is similar to that from previous studies.

**Table 2:** Class-wise performance score of the trained model.

Category	Name of anomaly	Precision	Sensitivity	Specificity
Soft tissue calcification	Calcified carotid atherosclerotic plaque	42.331	95.833	95.934
	Lymph node calcification	42.857	27.273	99.708
	Ossification of the stylohyoid ligament	56.391	95.541	96.784
	Tonsillar calcification	51.818	100.000	96.835
Carious lesions	Cervical caries or abrasion	51.625	76.744	95.160
	Dental caries or coronal defect	57.014	70.787	98.170
	Proximal caries	26.316	79.208	91.753
	Secondary caries	45.161	29.787	99.105
Anomalies in the dental region	External root resorption	40.000	33.333	99.345
	Impacted tooth	45.286	98.535	92.437
	Periapical radiolucency	47.817	95.292	89.514
	Residual root	35.017	90.435	97.243
	Supernumerary tooth	32.075	62.963	97.417
	Tooth overlapped with mandibular canal	53.759	100.000	93.237
Anomalies in surrounding region	Mucosal thickening in maxillary sinus	51.460	95.918	92.382
	Radiopacity in jaw	74.490	97.987	97.828
	Retention pseudocyst in maxillary sinus	61.538	92.308	99.042

**Table 3:** Class-wise performance score of the trained model.

Name of anomaly	Positive labeled objects	Predictions	True positives	Negative images	True negatives
Calcified carotid atherosclerotic plaque	72	126	69	1328	1274
Lymph node calcification	11	7	3	1370	1366
Ossification of the stylohyoid ligament	157	266	150	1275	1234
Tonsillar calcification	57	110	57	1327	1285
Cervical caries or abrasion	559	831	429	1095	1042
Dental caries or coronal defect	178	221	126	1257	1234
Proximal caries	202	608	160	1249	1146
Secondary caries	47	31	14	1341	1329
External root resorption	18	15	6	1374	1365
Impacted tooth	273	594	269	1190	1100
Periapical radiolucency	701	1397	668	906	811
Residual root	115	297	104	1306	1270
Supernumerary tooth	27	53	17	1355	1320
Tooth overlapped with mandibular canal	379	705	379	1109	1034
Mucosal thickening in maxillary sinus	147	274	141	1247	1152
Radiopacity in jaw	149	196	146	1243	1216
Retention pseudocyst in maxillary sinus	26	39	24	1357	1344

## Discussion

As we mentioned above, panoramic dental radiography is one of the most widely and frequently used imaging techniques in dentistry. It is safer than other imaging tools and quickly takes a wide range of dental structures so it is essential to make diagnoses and further treatment plans for patients, but due to the complicated structure of the dental region and lack of time, clinicians mainly focused on small parts of the images. If that neglected information is fully detected and noted to clinicians, it will improve the overall quality and consequences of the treatment. To achieve that goal, we applied an artificial intelligence technique which is widely used in the image analysis field to automatically detect regions of anomalies. We trained our model focused to increase the specificity to help clinicians filter out healthy panoramic dental radiography so as to decrease the number of images to be examined and to alleviate the burden of clinicians. First, we selected 17 major dental anomalies which are closely related to patients’ oral health. These anomalies can lead to serious outcomes if ignored or are related to other systemic diseases. It means that the early detection of those anomalies can prevent severe outcomes and can be used as a marker to suspect other systemic diseases. We categorized anomalies into four groups corresponding to their clinical features and locations.

First, carious lesions are considered the most prevalent problem in dentistry^14^. Because carious lesions cause more serious problems if ignored, prevention and early diagnosis are very important.

Many previous pieces of research applied artificial intelligence techniques to detect carious lesions^13^. Depending on its progress and location, various treatments can be used to treat carious lesions.

Here we divided carious lesions into four categories corresponding to their clinical features for fine-grained diagnoses; dental caries, cervical caries, proximal caries, and secondary caries. These subtypes of carious lesions show unique features which are related to their diagnoses and treatment. First, cervical caries is considered the most dangerous type because it leads to the rapid loss of tooth due to its location. Proximal caries is a type of carious lesion which is located on the surfaces between adjacent teeth. They are the most difficult type to detect because they cannot be visually or manually detected. Finally, secondary caries is a disease that occurs on the tooth after the filling. Because it takes a lot of burdens to detect^21^. This fine-grained diagnosis of carious lesions is important to early detection of caries before their progression to severe stages and to prevent further loss of dental tissues.

The second category of our fine-grained model, calcifications, occurs when calcium accumulates in body tissue. The diagnostic criteria of calcifications are their anatomical locations, distributions, numbers, sizes, and shapes^22^. Calcifications in maxillofacial areas can be found through examinations of panoramic dental radiography but there are very few studies conducted regarding them^23^. Though the presence of calcifications on panoramic dental radiographs is uncommon, their detection is important to prevent the further progression of diseases. We selected four calcification anomalies; Calcified carotid atherosclerosis plaque, lymph node calcifications, calcifications of the stylohyoid ligament, tonsillar calcifications (tonsilloliths)^24^.

Our third category is dental anomalies. We included dental disease features and abnormal structures shown in the dental region^18^ to this category. Dental anomalies are abnormal forms or structures of teeth in the dental area. We selected six dental anomalies which are critical factors of dental health; external root absorption, impacted tooth, periapical radiolucency, residual root, supernumerary tooth. Some of these anomalies often cause symptoms such as pain, halitosis, and bleeding, and can be used as diagnostic markers, and anatomical factors when planning further dental surgeries. For example, Periapical radiolucency is the radiographic changes around the apex of the tooth and is the sign of inflammatory bone lesions. Recent studies present that periapical radiolucency may be caused by several diseases such as cirrhosis^25^. External root resorption is an undesirable dental injury that causes a loss of some parts of a tooth and can be seen radiographically. This type of anomaly damages the underlying tissues and causes a number of complications including infection, loss of teeth, pain, and so on^26^. The positional relationship between the mandibular canal and corresponding tooth is a key anatomic factor to make surgical plans such as extraction of the mandibular third molar because damage to the inferior alveolar nerve affects the function of the stomatognathic system and the quality of life of patients^27^. Panoramic dental radiography is one way to evaluate the risk of nerve injury before the extraction^28^. Impacted teeth can cause several symptoms such as swollen gums, halitosis, and pain when opening the mouth. If ignored, it causes severe complications such as infection, cysts, absorption, and many gum diseases. A recent study presented that an impacted tooth might have some association with a large central osteoma^29^. A residual root is a leftover of a tooth in the jaw after an extraction. It sometimes causes infections and pain. Usually, it is recommended to extract with a local anesthetic. Finally, supernumerary teeth may lead to many severe problems like displacement, crowding, root resorption, dilaceration, loss of vitality of adjacent teeth, and even ameloblastomas and odontomas in severe cases. So, clinicians should aware of the existence of the occurrence so that they can formulate treatment plans^30^.

The last category is anomalies located in surrounding regions of the dental area. These anomalies are rarely related to oral health but may be used as potential markers to diagnose other related diseases. These radiographic anomalies are signs of inflammatory processes of that region and are known to be related to several diseases. For example, previous studies showed that retention pseudocysts of the maxillary sinus may have some associations with allergic and inflammatory processes, trauma, periapical and periodontal infections^31^, radiopacity in jaws with many osteoblastic and osteoclastic activities^32^, mucosal thickening of the maxillary sinus with apical periodontitis, alveolar bone loss, and so on^33^.

To train a deep learning model to detect these many types of anomalies, it is essential to accumulate datasets including enough number of objects for every class. In fact, the most important factor of using artificial intelligence techniques is the quality and quantity of data. We built the system to collect panoramic dental images directly from local dental clinics and manually labeled them by a dental radiography expert. For a year, we accumulated a large and high-quality dataset compared to previous studies. This dataset is also still growing, so it has great potential in this field. Our tool successfully detects given anomalies with high performance especially for specificity and demonstrates that artificial intelligence can reduce the burden of dental clinicians by reducing the number of images that should be examined manually through detecting potential anomalies and filtering normal images.

## Method

### Data acquisition and labeling

Images are taken from 30 local dental clinics by panoramic x-ray cameras model PaX-i, Rayscan alpha from VATECH, Papaya 3d from Genoray, and RealScan from PointNix. Images are fully anonymized removing all patient identifiers. We installed a data transfer module in imaging devices to automatically collect panoramic dental images from clinics. Images are collected from July 2020 to July 2021. During the data collection period, data collection and labeling anomalies are done in parallel. Ethics approval was not required for this study because panoramic dental image data used in this study is obtained fully anonymized and no identifiable private information is included, and the study is performed in accordance with the Declaration of Helsinki. For each image, above 17 anomalies are manually labeled one by one by a dental radiology expert. Significant features and diagnostics used to annotation of every anomaly are described in Table 1. Image regions showing features of anomalies are first labeled in box form and then in the polygonal form to specify the exact region of the anomalies. Supplementary table 1 contains detailed numbers of objects and images of every anomaly.

### Model architecture and training

Because images are originally transferred in DICOM format, we converted images to PNG format. We used faster R-CNN architecture for dental anomaly detection^19^. We trained a model for every 17 dental anomalies. We divided our dataset into a train/validation set and a test set. All images taken from July 2020 to March 2021 are used as a train/validation set, and others which are taken from April 2021 to July 2021 are used as a test set. We built a position verifier to filter out wrongly detected object boxes from the R-CNN model. It takes each object class and its location and examines whether it is well detected or not. Two classes, Carious lesions and anomalies in the dental region, are expected to be in the dental region and other classes are expected to be in the surrounding region. Finally, in the polygon shaper stage, we used Detectron2^20^ to narrow down the detected regions from box form to polygon shape.

### Detection and evaluation

To evaluate our model, we used an intersection over union (IOU) value to determine correctly-detected lesions from the output of the model. IOU is a widely used metric to evaluate the performance of object detection models by calculating the intersection over union between the ground-truth area and the prediction area. We determined the predicted box is correctly-detected when its IOU value is over 0.5. After the detection, we calculated precision, sensitivity, and specificity for evaluation. Table 3 shows detailed numbers of detection result on test dataset.

## Supplementary Information

Supplementary Table 1.
