# Supplementary material for: Detecting 17 fine-grained dental anomalies from panoramic dental radiography using artificial intelligence
Source: Sci Rep. 2022 Mar 25;12:5172. doi: 10.1038/s41598-022-09083-2 (PMC8956729; doi:10.1038/s41598-022-09083-2)
Supplement: Supplementary file 1 — Supplementary Table 1. [file 41598_2022_9083_MOESM1_ESM.pdf]

Detecting 17 fine-grained dental anomalies from panoramic dental radiography using artificial intelligence

Sangyeon Lee, Donghyun Kim, Hogul Jeong

Supplementary information

Supplementary table 1. Overall numbers of training and validation datasets

**Anomalies**	**Number of positive objects, training**	**Number of positive images, training**	**Number of positive objects, validation**	**Number of positive images, validation**
Calcified carotid atherosclerotic plaque	634	496	137	112
Lymph node calcification	139	124	32	29
Ossification of the stylohyoid ligament	1,917	1,271	169	124
Tonsillar calcification	545	467	109	100
Cervical caries or abrasion	5,384	2,656	503	356
Dental caries or coronal defect	2,776	1,895	423	263
Proximal caries	2,725	1,527	1,230	657
Secondary caries	707	586	153	122
External root resorption	215	140	45	26
Impacted tooth	1,715	1,305	413	298
Periapical radiolucency	5,054	3,471	1,358	863
Residual root	1,050	678	267	171
Supernumerary tooth	434	394	84	71
Tooth overlapped with mandibular canal	3,123	2,143	662	483

Mucosal thickening in maxillary sinus	1,227	1,095	311	284
Radiopacity in jaw	702	655	177	167
Retention pseudocyst in maxillary sinus	131	128	24	24
